# Piloting a Community Education Skin Cancer Program Coordinated by Medical Students

**DOI:** 10.2196/36793

**Published:** 2022-08-11

**Authors:** Rewan Abdelwahab, Maya Abdou, Catherine Newman

**Affiliations:** 1 Mayo Clinic Alix School of Medicine Rochester, MN United States; 2 Department of Dermatology Mayo Clinic Rochester, MN United States

**Keywords:** skin cancer, skin, cancer, oncology, dermatology, community education, health education, pediatric, paediatric, skin of color, sun exposure, tanning, service, child, school age, school, student, medical student, health literacy, telehealth, teledermatology, telemedicine, online education, distance education, internet-based, digital health, melanoma, patient education, prevention

Skin cancer is the most common cancer in the United States with a dramatic increase in the risk of melanoma development after serious sunburn [1]. The United States Preventative Service Task Force recommends that all children and young adults aged 6 months to 24 years be counseled about skin cancer prevention and sun protective habits [2]. Block the Blaze (BTB) is a nonprofit run through the John Wayne Cancer Foundation (JWCF) that is dedicated to educating school-age students about skin cancer detection and sun-protective habits. The Mayo Clinic Alix School of Medicine (MCASOM) is one of the few medical school chapters of the nonprofit, highlighting opportunities for increased medical student community engagement. All volunteers were required to complete both the Melanoma Research Foundation’s melanoma educator certification course and virtual training held by the JWCF on presenting to school-age children in the community. Community presentations focus on teaching school-age children about sun-protective habits, skin cancer risk factors, and completing thorough skin checks. The goal of the MCASOM BTB community education program is to reach as many students as possible virtually while working to transition to a hybrid virtual and in-person format and expanding within the Rochester Public School District.

The leadership structure for BTB at MCASOM consists of an internal team that coordinates volunteers and an external team that coordinates community outreach for presentations. Presentations can only be scheduled during the high school, middle school, and elementary school academic calendar from September to May.

Although leadership for BTB consisted of students from Mayo Clinic campuses in Arizona and Minnesota in the first half of its term, the 2 volunteer groups began to run autonomously in the second half of its term in anticipation of in-person community presentations as the pandemic subsides. The timelines of the first and second terms are visualized in Figures 1 and 2.

Modifications to the curriculum provided by the JWCF included updating California skin cancer statistics to those of the local state. Images were also updated to include more skin of color. Photographs of dermatological conditions on skin of color are limited and variable at the national level [3], which in turn can be contributing to survival rate disparities and delayed diagnosis of melanoma in people of color [4].

A total of 113 teachers were contacted in Minnesota during the first term across different middle and high schools. The response rate to external emails was around 3.5%. A total of 40 presentations were scheduled, and 424 students were reached at local schools, representing more than 2.4% (424/17,474) of Olmsted County’s student population. The virtual programming facilitates greater geographic reach for contacted schools and provides scheduling flexibility so that volunteers from other campuses could cover presentation shifts in a different state when needed. However, the drawbacks of the virtual format include technical and connection problems, lack of audience feedback, and difficulties in coordinating with schools that are having in-person classes. Community engagement interventions are often used to improve public health awareness and education, address health care disparities, and offer social support for disadvantaged groups [5].

**Figure 1:**
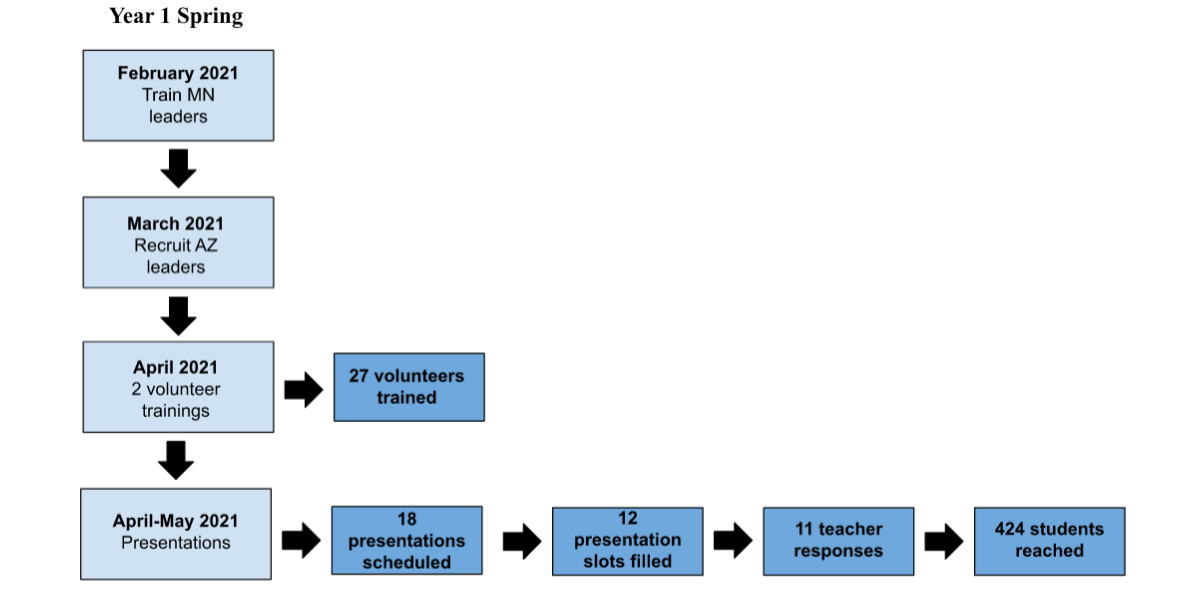
Framework, timeline, and results of the first half of year 1 (spring). AZ: Arizona, MN: Minnesota.

**Figure 2:**
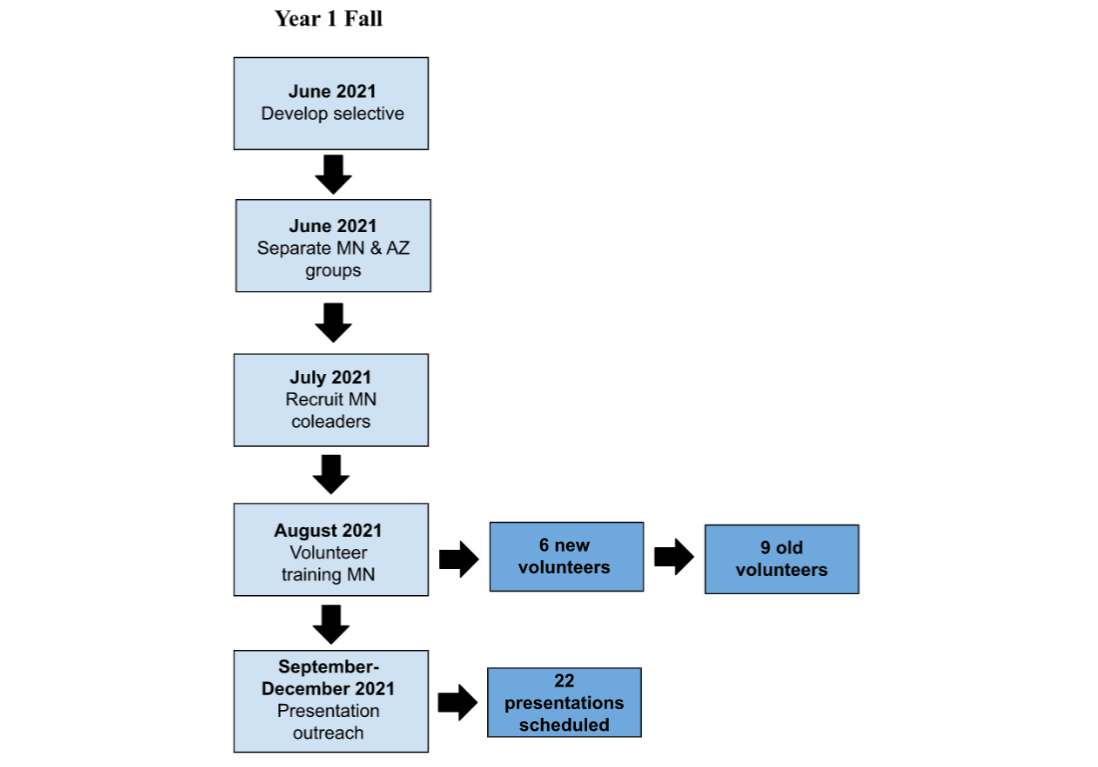
Framework, timeline, and results of the second half of year 1 (fall). AZ: Arizona, MN: Minnesota.

## Abbreviations

BTB: Block the Blaze
JWCF: John Wayne Cancer Foundation
MCASOM: Mayo Clinic Alix School of Medicine

## References

[1] Dowd M Denise (2019). Treatment and prevention of pediatric sunburn. Pediatr Ann.

[2] Skin Cancer Prevention: Behavioral Counseling (2018). Skin cancer prevention: behavioral counseling. United States Preventive Services Taskforce.

[3] Amuzie Adaure U, Jia Justin L, Taylor Susan C, Lester Jenna C (2022). Skin-of-color article representation in dermatology literature 2009-2019: Higher citation counts and opportunities for inclusion. J Am Acad Dermatol.

[4] Zakhem GA, Pulavarty AN, Lester JC, Stevenson ML (2022). Skin cancer in people of color: a systematic review. Am J Clin Dermatol.

[5] O'Mara-Eves A, Brunton G, McDaid D (2013). Community Engagement to Reduce Inequalities in Health: A Systematic Review, Meta-Analysis and Economic Analysis.

